# Assessing food security performance from the One Health concept: an evaluation tool based on the Global One Health Index

**DOI:** 10.1186/s40249-023-01135-7

**Published:** 2023-09-22

**Authors:** Si-Yu Gu, Fu-Min Chen, Chen-Sheng Zhang, Yi-Bin Zhou, Tian-Yun Li, Ne Qiang, Xiao-Xi Zhang, Jing-Shu Liu, Shu-Xun Wang, Xue-Chen Yang, Xiao-Kui Guo, Qin-Qin Hu, Xiao-Bei Deng, Le-Fei Han

**Affiliations:** 1 School of Global Health, Chinese Center for Tropical Diseases Research, Shanghai Jiao Tong University School of Medicine, Shanghai, 200025 China; 2 One Health Center, Shanghai Jiao Tong University-The University of Edinburgh, Shanghai, 200025 China; 3 National Institute of Parasitic Diseases, Chinese Center for Disease Control and Prevention (Chinese Center for Tropical Diseases Research), Shanghai, 200025 China; 4 Minhang District Center for Disease Control and Prevention, Shanghai, 201101 China; 5 School of Public Health, Shanghai Jiao Tong University School of Medicine, Shanghai, 200025 China

**Keywords:** Food security, One Health Index, Indicator framework, Assessment

## Abstract

**Background:**

Food systems instantiate the complex interdependencies across humans, physical environments, and other organisms. Applying One Health approaches for agri-food system transformation, which adopts integrated and unifying approaches to optimize the overall health of humans, animals, plants, and environments, is crucial to enhance the sustainability of food systems. This study develops a potential assessment tool, named the global One Health index-Food Security (GOHI-FS), aiming to evaluate food security performance across countries/territories from One Health perspective and identify relevant gaps that need to be improved for sustainable food systems.

**Methods:**

We comprehensively reviewed existing frameworks and elements of food security. The indicator framework of GOHI-FS was conceptualized following the structure-process-outcome model and confirmed by expert advisory. Publicly available data in 2020 was collected for each indicator. The weighting strategy was determined by the Fuzzy Analytical Hierarchy Process. The data for each indicator was normalized and aggregated by weighted arithmetic mean. Linear regressions were performed to evaluate the associations of GOHI-FS with health and social-economic indicators.

**Results:**

The GOHI-FS includes 5 first-level indicators, 19 second-level indicators and 45 third-level indicators. There were 146 countries/territories enrolled for evaluation. The highest average score of first-level indicators was Nutrition (69.8) and the lowest was Government Support and Response (31.3). There was regional heterogeneity of GOHI-FS scores. Higher median scores with interquartile range (IQR) were shown in North America (median: 76.1, IQR: 75.5–76.7), followed by Europe and Central Asia (median: 66.9, IQR: 60.1–74.3), East Asia and the Pacific (median: 60.6, IQR: 55.5–68.7), Latin America and the Caribbean (median: 60.2, IQR: 57.8–65.0), Middle East and North Africa (median: 56.6, IQR: 52.0–62.8), South Asia (median: 51.1, IQR: 46.7–53.8), and sub-Saharan Africa (median: 41.4, IQR: 37.2–46.5). We also found significant associations between GOHI-FS and GDP per capita, socio-demographic index, health expenditure and life expectancy.

**Conclusions:**

GOHI-FS is a potential assessment tool to understand the gaps in food security across countries/territories under the One Health concept. The pilot findings suggest notable gaps for sub-Saharan Africa in numerous aspects. Broad actions are needed globally to promote government support and response for food security.

**Supplementary Information:**

The online version contains supplementary material available at 10.1186/s40249-023-01135-7.

## Background

In food system, the complex relationship across humans, animals, plants, and ecosystems may lead to fuzzy and fragmented oversight or governance for some food-related issues. Maintaining sustainable food security is an important guarantee for promoting economic growth, human’s well-being and social harmony [1, 2]. It is also important for eliminating hunger and achieving Sustainable Development Goals (SDG) [3]. Although there have been substantial efforts to maintain food security, various threats from human–animal–plant-environment interfaces raise complex challenges in food system [4]. For instance, emerging zoonotic viruses [e.g., severe acute respiratory syndrome coronavirus (SARS-CoV), Nipah, Ebola, SARS-CoV-2] can spill out to humans via food systems [5]. Unrestricted land reclamation can lead to resource depletion, soil erosion, environmental pollution, etc. [1], which in turn leads to a decline in food production. Climate change and natural disasters can undermine food production and be associated with emerging zoonosis [6, 7]. In addition, overgrazing, the wide use of antibiotics and pesticides, and heavy metal pollution can also threaten the health of animals or plants, and further affect the safety of food from farm to fork.

To identify possible solutions for the challenges in such a complex system, a One Health approach has been proposed as a feasible way to achieve food security via adopting integrated and unifying approaches to optimize the overall health of people, animals, plants, and ecosystems [8]. As a key agency that leads international efforts to defeat hunger and achieve food security, the Food and Agriculture Organization of the United Nations (FAO) is actively promoting One Health approaches for agrifood transformation with cooperative efforts from the United Nations Environment Programme (UNEP), the World Health Organization (WHO) and the World Organization for Animal Health (WOAH) [9]. The implementation of One Health approaches in the field of food security is meaningful. It may better balance environmental problems and directly increase the yield and quality of food. It may also benefit zoonotic diseases control and prevention in food systems, improve food productivity, and reduce the risk of food biosafety caused by zoonosis [10, 11].

To understand the global situations of food security under the One Health concept, it is important to have a comprehensive evaluation tool to assess the One Health performance worldwide. Although there have been several evaluation tools in regard to food security/safety, such as global food security index (GFSI), global hunger index (GHI) and food sustainability index (FSI), few of them were developed based on the One Health concept. To fill this gap, the global One Health index (GOHI) was developed to assess the One Health performance across countries/territories with multiple sources of data from authoritative databases [12]. The framework of GOHI was cell-like that contained three components, including external issues on social and economic factors, intrinsic issues on human, animal and environmental health, and core issues of One Health including zoonotic disease, food security, antimicrobial resistance (AMR), climate change, and governance. Among them, food security related indicators and the evaluation framework were included as a part of GOHI, named global One Health index-Food Security (GOHI-FS).

In this study, we described the conceptual framework of GOHI-FS. By matching and synthesizing key elements of GOHI-FS with qualitative and quantitative data from multiple authoritative sources, we established the GOHI-FS database and performed a pilot analysis to understand the global performance of food security with a One Health concept. The results may help identify gaps of achieving food security and sustainable food system at regional and national levels and provide suggestions for improvement.

## Methods

### Framework formulation

To develop the indicator framework of GOHI-FS, we considered the core pillars of food security proposed by High-Level Panel of Experts on Food Security and Nutrition (HLPE-FSN) in 2020 as the first-level indicators [13]. The six pillars were availability, access, utilization, stability, agency, and sustainability, which emphasized the coordinated development of human–animal-environment systems as part of the agrifood system transformation. We integrated the concept of six pillars into the five first-level indicators of GOHI-FS as Food Demand and Supply, Food Safety, Nutrition, Natural and Social Circumstances, and Government Support and Response. Of them, Food Demand and Supply assessed the availability, access, and stability for food production and demand. Food Safety assessed multiple components associated with keeping food safe, including agency, policy, regulation as well as the disease burden of foodborne related causes. Nutrition is the indicator that evaluated utilization of food. Natural and Social Circumstances considered the sustainability of the natural and social environment related to food system. The indicator of Government Support and Response evaluated the involvement and initiative of government entities in capacity building for strengthening the resilience of the food system.

The second-level indicators were designed to evaluate the corresponding first-level indicators from the structure, process and outcome perspectives followed a structure-process-outcome (SPO) model [14]. The third-level indicators and matched data were collected from multiple authoritative databases (i.e., FAO, WHO, United Nations (UN), etc.) and were subsequently reviewed for relevance and data availability.

### Data collection and indicator selection

All the indicators of GOHI-FS were selected in accordance with the principles of authoritative sources, relevance, open access, timeliness, completeness, comparability, and country-level data. The expert advisory committee with 29 experts of human health, veterinary science, environmental science, and social science also conducted several rounds of consultations to ensure the reliability and validation of the GOHI-FS framework, indicators, and matched data. We also performed several interviews with experts from authoritative agencies, such as UN, FAO, WHO, and World Bank, to reach agreement for indicator scheme and match available data for evaluation.

We collected publicly available data for GOHI-FS database construction. Quantitative data were extracted from the authoritative agencies, including FAO, WHO, UN, World Bank, United Nations High Commissioner for Refugees (UNHCR), United Nations Environment Programme (UNEP), etc. For qualitative data, some were assigned to ordered categorical values or discrete values according to textual information provided in national annual reports, structured surveys, or open-access authoritative sources. All the data were retrieved in November 2021. For each indicator, the most recent data when available was used for analysis. To ensure the quality of the data, we double checked the validity, consistency, and quality during data collection. The data source and the information of each three-level indicator can be found in Additional file 1.

### Weight determination

We used Fuzzy Analytical Hierarchy Process (FAHP) to determine weights for most of the indicators [15]. During this process, we conducted two rounds of interrogation to collect experts’ opinions on the relative importance judgments between each pair of two indicators by questionnaires. We used fuzzy comparison matrix based on different experts’ judgments to generate the weight matrix of indicators. The details of expert interrogation and weight determination can be found in our previous publication [12]. For the third-level indicators, equal weights were assigned to those indicators. The final weight scheme was confirmed by the expert advisory committee.

### Data processing and statistical analysis

We collected data for 220 countries/territories if data were available. Countries/territories were excluded if over 50% data were missed in the GOHI database. The threshold of 50% missing rate was set given broad data incompleteness among countries/territories and we would like to enroll more areas to understand the progress around the world. Indicators were excluded from analysis if relevant data was absent in more than 160 countries/territories. A total of 146 countries/territories were included from 7 world regions. The missing data were interpolated by multiple imputation and controlled by GDP, Human Development Index (HDI), and life expectancy to balance the disparities from economy, social development, and health status.

To ensure data comparability across indicators, all the quantitative and qualitive data were rescaled within a range of 0–100. We assumed a higher score represent a better performance for each indicator. All the quantitative data were checked for normality and log-transformed if they were skewed. To remove outliers, we set best/worst value and rescaled the data with variation between 2.5th percentile and 97.5th percentile of the raw data. For some indicators that have clear range or clear goals to achieve, the certain best/worst values were set according to the values. After indicating the best/worst value of each indicator, the raw data were transformed linearly through following equation to normalize the raw data between 0 and 100 [12, 16]:

$$S=({X-{X}_{worst})/(X}_{best}-{X}_{worst})\times 100$$

where *S* denotes the normalized score for indicators of countries/territories; $X$ denotes the raw values for the indicator of countries/territories; ${X}_{best}$ denotes the value of best performance for the indicator; ${X}_{worst}$ denotes the value of worst performance for the indicator.

The weighted sum scores of the lower-level indicators were calculated according to the normalized values and corresponding indicator weights through the following equation to obtain scores of upper-level indicators:

$${indicator}\;{score}_{ih}=\sum_{{1}_{h}}^{{m}_{h}}{S}_{{in}_{h}}\times {W}_{{n}_{h}}, \sum_{{1}_{h}}^{{m}_{h}}{W}_{{n}_{h}}=1$$

where ${m}_{h}$ denotes the total number of lower-level indicators under the h-th upper-level indicator; ${n}_{h}$ denotes the n-th lower-level indicator under the h-th upper-level indicator; ${S}_{{in}_{h}}$ denotes the score of ${n}_{h}$-th indicator of the i-th country; ${\text{W}}_{{\text{n}}_{\text{h}}}$ denotes the weight of $\mathrm{n}$-th indicator.

The collinearity across indicators was assessed by Spearman correlation coefficients. Indicators with high coefficients (*r* > 0.7 or *r* < − 0.7) were carefully discussed on whether to remove or retain according to different aspects that need to be assessed.

We performed stratification analysis by geographic region and social demographic state to explore the regional and social development disparities. We used World Bank standard country coding for 7 world regions. There were 19 countries from East Asia and Pacific, 47 countries from Europe and Central Asia, 18 countries from Latin America and the Caribbean, 16 countries from Middle East and North Africa, 2 countries from North America, 7 countries from South Asia and 37 countries from sub-Saharan Africa (Additional file 2). The enrolled countries were also classified into 5 levels according to Socio-demographic Index (SDI, a combined information about GDP per capita, average years of schooling among individuals under 25 years, and total fertility rate among females under 25 years) [17], including 31 high SDI countries, 36 high-middle SDI countries, 28 middle SDI countries, 26 low-middle SDI countries, and 25 low SDI countries (Additional file 3).

To understand the application of GOHI-FS and its association with national socioeconomic and health status, we also performed linear regression to explore the associations between GOHI-FS and other social, economic and health factors, including SDI, Gross Domestic Product per capita (GDP), Current health expenditure (CHE), average life expectancy (Exp). All the data processing and statistical analysis were performed using **R** software (version 4.1.2, Posit Software, Vienna, Austria), packages mainly included dplyr, ggplot2, ggpairs and other basic functions.

## Results

### Indicator framework

**Fig. 1:**
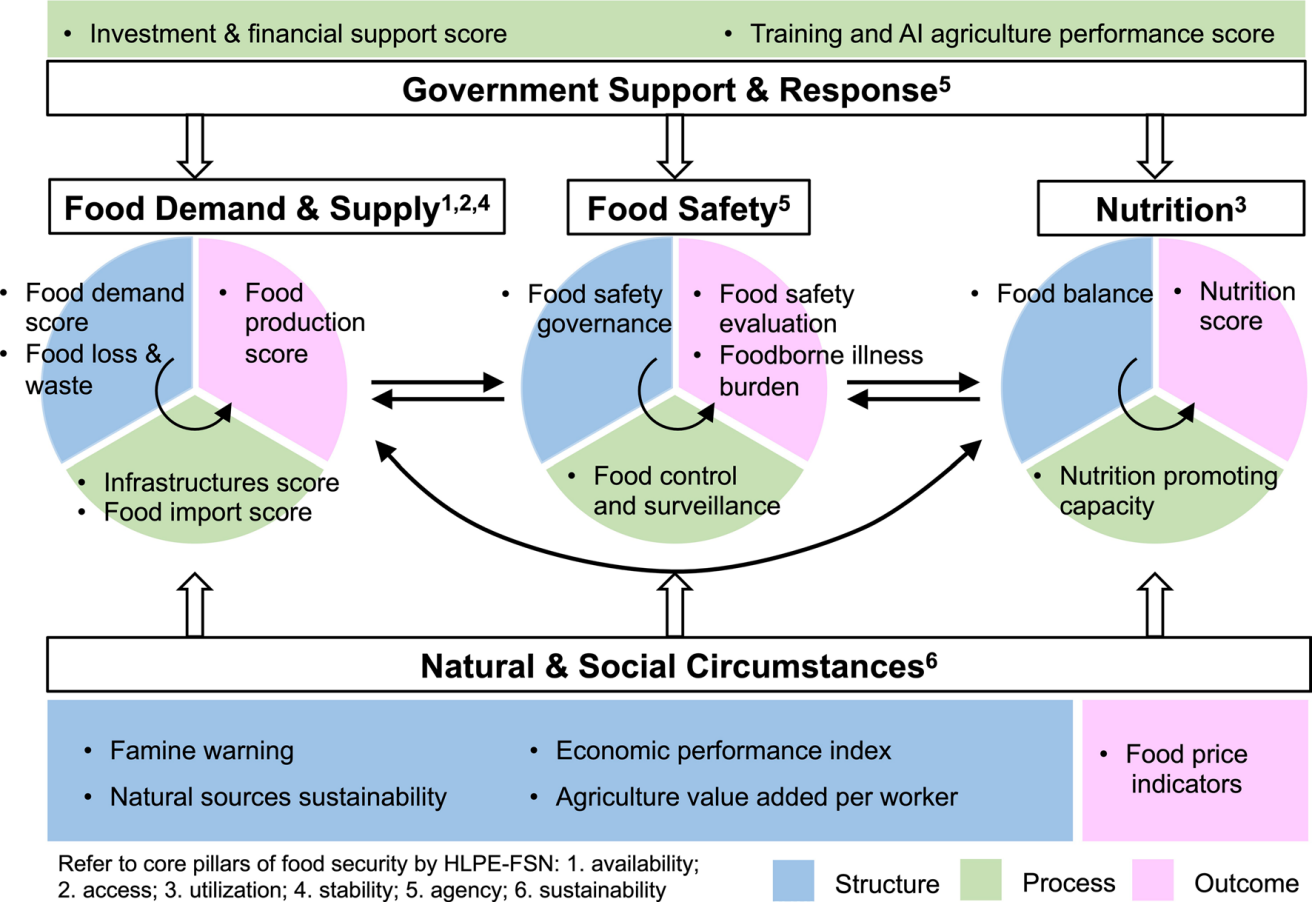
Indicator framework of global One Health index-Food Security

The GOHI-FS framework includes 5 first-level indicators, 19 second-level indicators, and 45 third-level indicators that assess the performance of food security from One Health perspective. The complete indicator framework is shown in Fig. 1 and Table 1. The Spearman correlation coefficients across the second-level indicators and the third-level indicators were shown in Additional files 4 and 5. Overall, the coefficients were low to moderate across most of the same-level indicators.

**Table 1:** Indicators and weight scheme of global One Health index-Food Security

Categories	Weight (%)	Key indicators	Type^a^	Weight (%)	Indicators	Weight (%)
Food demand and supply	20.00	1.1 Food demand score	s	21.8	1.1.1 Ratio of population growth	40.0
					1.1.2 Ratio of refugees and internally displaced people	20.0
					1.1.3 Ratio of moderately or severely food insecure people	40.0
		1.2 Food loss and waste	s	20.2	1.2.1 Food loss	50.0
					1.2.2 Food waste	50.0
		1.3 Infrastructures score	p	19.4	1.3.1 Logistic performance index	33.3
					1.3.2 Net capital stocks	33.3
					1.3.3 Percent of arable land equipped for irrigation	33.3
		1.4 Food import score	p	14.7	1.4.1 Cereal import dependency ratio	33.3
					1.4.2 Value of food imports over total merchandise exports	33.3
					1.4.3 Food aid	33.3
		1.5 Food production score	o	23.9	1.5.1 Average value of food production	50.0
					1.5.2 Food production viability	50.0
Food safety	20.00	2.1 Food safety governance	s	30.3	2.1.1 Food safety agency	50.0
					2.1.2 Food policy, legal and regulatory framework	50.0
		2.2 Food control and surveillance	p	26.7	2.2.1 Inspections in farm-to-fork food chain	50.0
					2.2.2 Food recalls	50.0
		2.3 Food safety evaluation	o	22.4	2.3.1 Food safety score	100.0
		2.4 Foodborne illness burden	o	20.6	2.4.1 Disability-Adjusted Life Years of diarrhea	100.0
Nutrition	20.00	3.1 Food balance^s^	s	39.3	3.1.1 Average dietary energy supply adequacy	33.3
					3.1.2 Average protein supply	33.3
					3.1.3 Per capita food supply variability	33.3
		3.2 Nutrition promoting capacity	p	30.1	3.2.1 Nutrition labeling	33.3
					3.2.2 Nutrition guideline	33.3
					3.2.3 Nutrition education programme	33.3
		3.3 Nutrition score	o	30.6	3.3.1 Undernourishment	33.3
					3.3.2 Stunting in children under five	33.3
					3.3.3 Anemia among women of reproductive age	33.3
Natural and social circumstances	20.00	4.1 Famine warning	s	22.6	4.1.1 Food affected by extreme weather conditions, disasters, or crisis	100.0
		4.2 Natural sources sustainability	s	24.9	4.2.1 Per person land under cereal production	20.0
					4.2.2 Agricultural water withdrawal as % of total renewable water resources	20.0
					4.2.3 Agriculture area under organic agric	20.0
					4.2.4 Naturally regenerating forest	20.0
					4.2.5 Manure management	20.0
		4.3 Economic performance index	s	18.6	4.3.1 Trade balance indicators	50.0
					4.3.2 Economic vulnerability index	50.0
		4.4 Agriculture value added per worker	s	18.2	4.4.1 Agriculture value added per worker	100.0
		4.5 Food price indicators	o	15.7	4.5.1 Agricultural import tariffs	33.3
					4.5.2 Consumer prices food indices	33.3
					4.5.3 Food price inflation	33.3
Government support and response	20.00	5.1 Investment and financial support score	p	55.4	5.1.1 Government investment on agriculture	33.3
					5.1.2 Credit to agriculture, forestry, and fishing	33.3
					5.1.3 R&D Expenditures	33.3
		5.2 Training and AI agriculture performance score	p	44.6	5.2.1 Training programme	50.0
					5.2.2 Smart and digital agriculture	50.0

^a^ According to the “structure-process-outcome” model, the indicators are divided into different categories: “s” represents “structure” that is resource allocation, “p” represents “process” that is intervention, and “o” represents “outcome” that is performance after intervention

### Food security performance measured by GOHI-FS

**Fig. 2:**
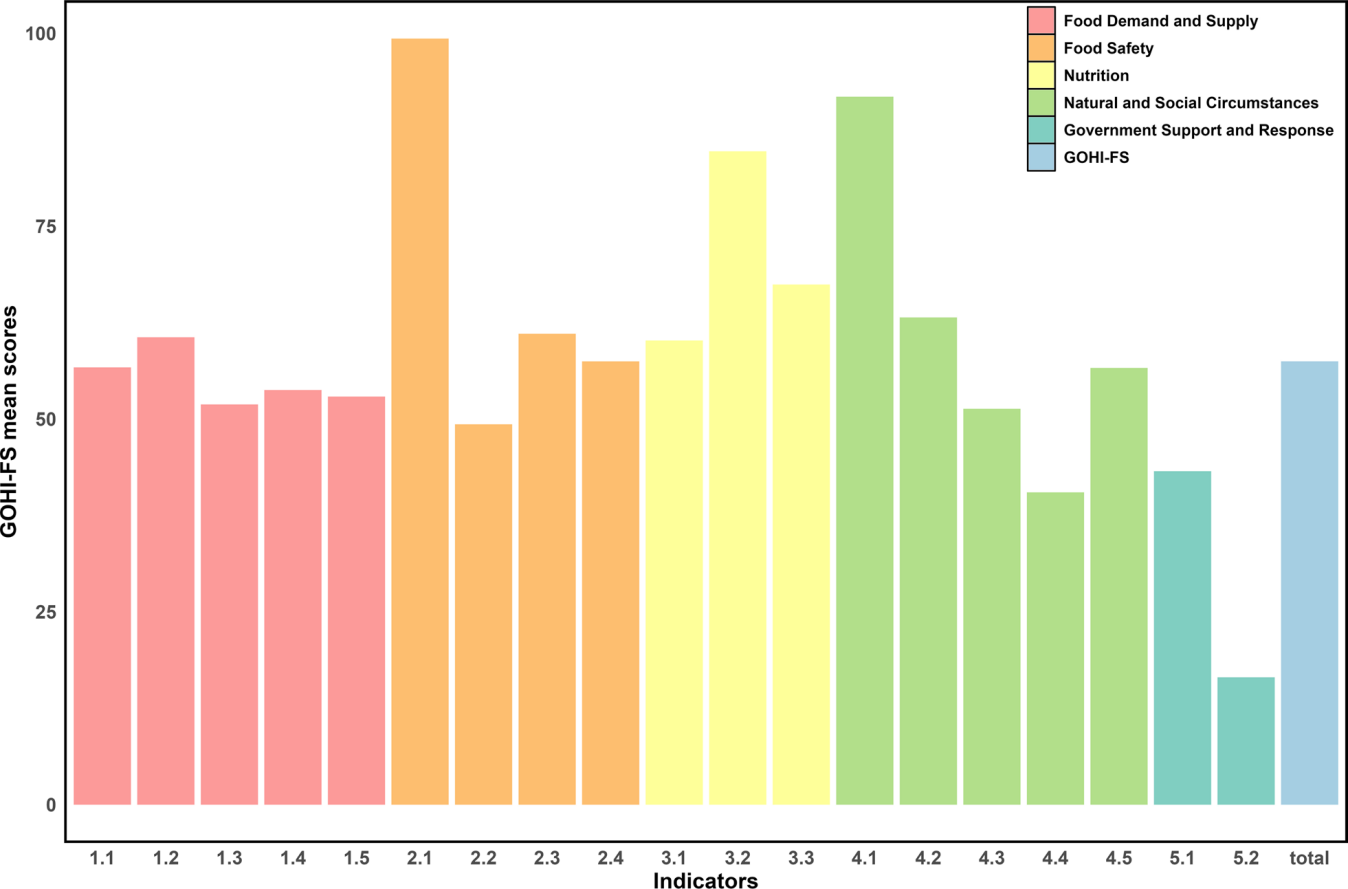
Average scores of global One Health index-Food Security (GOHI-FS) by second-level indicator

According to our statistical protocol, a higher total score indicates a relatively better food security performance compared with other countries/territories. The median score with interquartile range (IQR) of the total GOHI-FS scores among 146 countries/territories in 2020 is 59.0 (IQR: 48.1–66.9). The highest GOHI-FS score is 78.6 (Switzerland and Australia) and the lowest score is 29.5 (Central African Republic) (Additional file 6). The median scores of the first-level indicators are 57.0 (IQR: 46.7–64.7) for Food Demand and Supply, 66.9 (IQR: 53.3–88.9) for Food Safety, 70.8 (IQR: 57.5–83.6) for Nutrition, 63.3 (IQR: 58.2–69.7) for Natural and Social Circumstances, and 31.2 (IQR: 25.2–38.8) for Government Support and Response. The average scores of the 19 second-level indicators are shown in Fig. 2.

### Regional performance of GOHI-FS

The range of GOHI-FS score among 146 countries/territories are shown in Additional file 7. The median (IQR) scores were 76.1 (IQR: 75.5–76.7) in North America, 66.9 (IQR: 60.1–74.3) in Europe and Central Asia, 60.6 (IQR: 55.5–68.7) in East Asia and the Pacific, 60.2 (IQR: 57.8–65.0) in Latin America and the Caribbean, 56.6 (IQR: 52.0–62.8) in Middle East and North Africa, 51.1 (IQR: 46.7–53.8) in South Asia, and 41.4 (IQR: 37.2–46.5) in sub-Saharan Africa. Among the 25 countries/territories with scores over 70, 19 are from Europe and Central Asia, three from East Asia and the Pacific, two from North American and one from Latin America and the Caribbean. Among the 17 countries/territories with scores less than 40, 16 are from sub-Saharan Africa and the rest one is from South Asia.

**Fig. 3:**
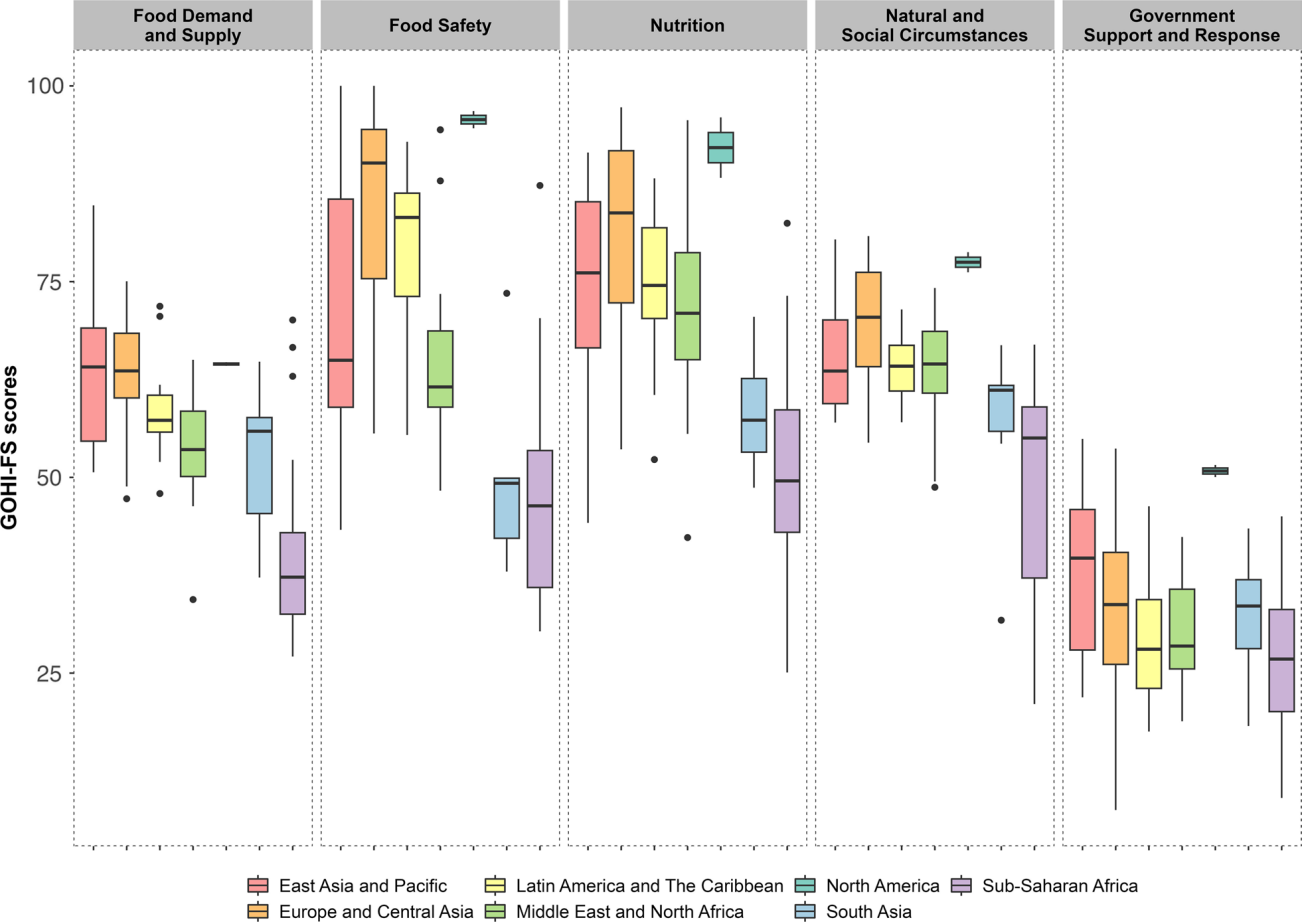
Boxplots of global One Health index-Food security scores by first-level indicator across regions. The length of the box is the interquartile interval, and the horizontal line is the median score in each region

The regional heterogeneity is also shown on the first-level indicators of GOHI-FS (Fig. 3). North America has higher scores across the five first-level indicators than other regions. For Food Supply and Demand, most countries/territories had scores centered between 46.0 and 64.7. East Asia and the Pacific, Europe and Central Asia, and North America had similar median scores about 63–64. Sub-Saharan Africa has the lowest median of 37.2 (IQR: 32.5–42.9). For Food Safety, the highest median score is shown in North America (median: 95.7, IQR: 95.1–94.4), followed by Europe and Central Asia (median: 90.1, IQR: 75.4–94.4), Latin America and the Caribbean (median: 83.2, IQR: 73.1–86.3), East Asia and the Pacific (median: 64.4, IQR: 58.1–77.5), Middle East and North Africa (median: 61.5, IQR: 59.0–68.7), South Asia (median: 49.2, IQR: 42.2–49.8), and sub-Saharan Africa (median: 46.4, IQR: 35.9–53.4). For Nutrition, 75% countries/territories in East Asia and Pacific, Europe and Central Asia, Latin American and the Caribbean, Middle East and North Africa, and North American had scores over 70. The median in South Asia and sub-Saharan African is 51.2 (IQR: 43.1–58.6). The regional heterogeneity of natural and social circumstance is less clear, except for some regions in sub-Saharan African which have lower scores. Due to the data restriction, Government Support and Response of each region had the lowest scores than other first-level indicators. North America had relatively better performance than other regions, followed by East Asia and Pacific. The scores in other regions had similar ranges from 7.5 to 53.7.

### Associations between GOHI-FS and other health and social-economic indicators

**Fig. 4:**
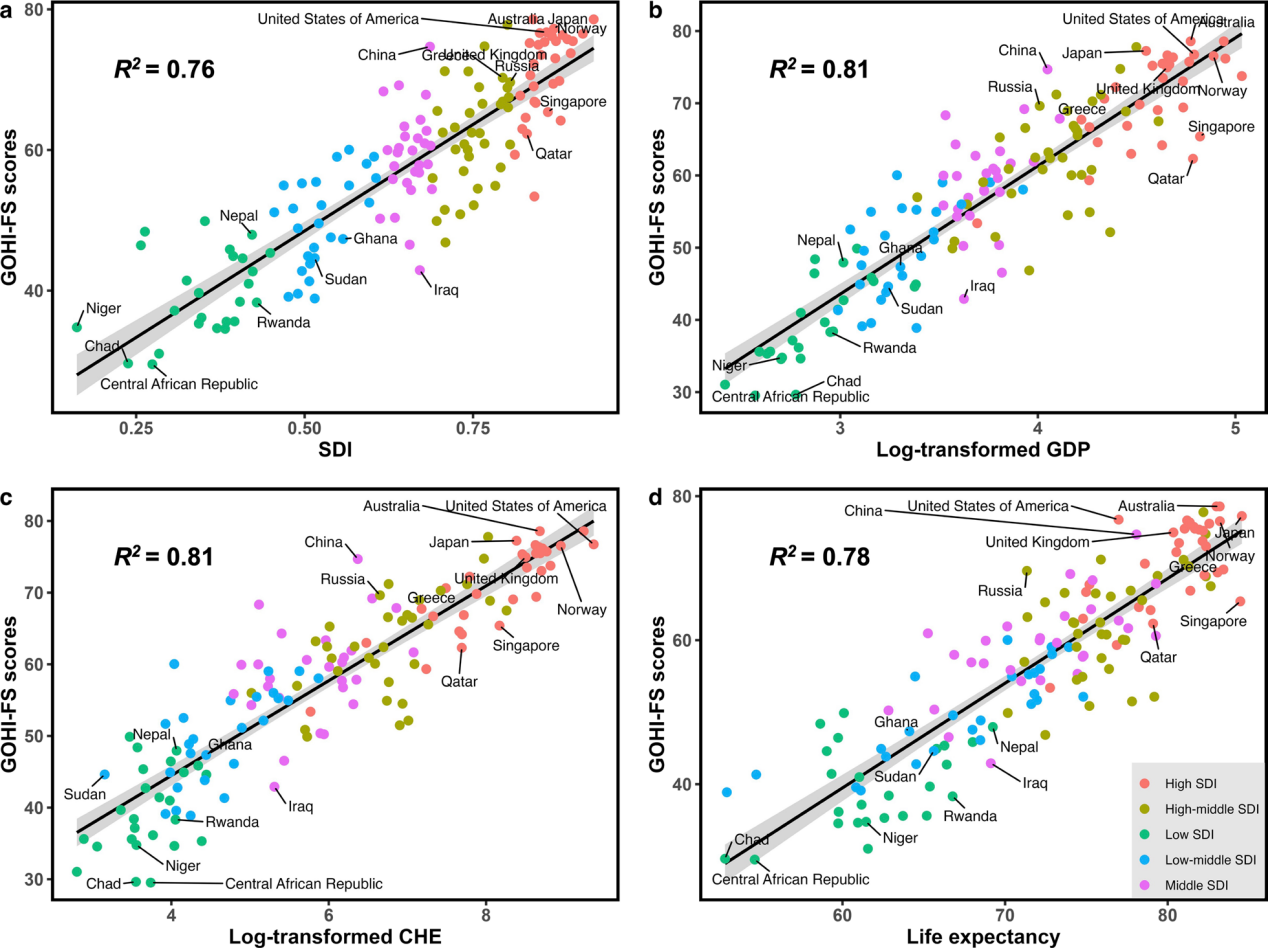
Associations of global One Health index-Food security scores with **a** social-demographic index, **b** log-transformed GDP per capita in USD, **c** log-transformed current health expenditure per capita in USD, and **d** life expectancy. *CHE* current health expenditure, *GDP* Gross Domestic Product, *SDI* Social-Demographic Index, *USD* United State Dollars. Dots in different colors indicates countries/territories in different SDI levels, refer to Additional file 3 for details

We used linear regression models to compare the associations of GOHI-FS among SDI, GDP per capita, CHE and EXP (Fig. 4). The results show GOHI-FS is positively associated with those health and social-economic indicators. Specifically, the correlation coefficients (*R*^2^) between GOHI-FS and SDI are 0.76, 0.81 for logged-transformed GDP per capita, 0.81 for logged-transformed CHE, and 0.78 for life expectancy. *P*-values for all coefficients are less than 0.001.

## Discussion

In this study, we introduced an evaluation tool, GOHI-FS, as a part of the global One Health index to evaluate the performance of food security across countries/territories. GOHI-FS integrated multiple indicators related to food security and developed the evaluation framework from a holistic perspective of One Health. Our pilot analysis showed the global food security performance are far from optimal.

### Uniqueness of GOHI-FS

There have been several existing indicators developed by academic institutions, national governments, and international agencies to measure or monitor the sustainable development, overall performance, or progress of food security. The GHI aims to measure “hunger” using four equally weighted indicator framework with a purpose of “highlight successes and failures in hunger reduction” and “raise awareness and understanding of regional and country differences in hunger” [18]. Designed by the Economist Intelligence Unit, the GFSI is another multi-dimensional tool for assessing country-level trends in food security with four dimension about availability, access, utilization and stability [19]. FSI aims to evaluate food security based on the three dimensions of food loss and waste, sustainable agriculture, and nutrition challenges [20]. Cleveland et al. discussed food security indicators should be specific to spatial scale and be adapted to the local natural, social, and economic environment [21]. Although several indicator frameworks have been investigated, further refinements on the healthy and sustainable food security should embrace the concept of One Health, since it is necessary to solve the more complex food security problems effectively from a more comprehensive and integrated perspective [4].

The GOHI-FS index framework was developed with the identification the complexed interaction across human, animal, plant, ecosystem, and social environment and a possible solution via One Health practice in the food security field. The conceptual framework of the first-level indicators was in line with the definition of food security by FAO as “Food security exists when all people, at all times, have physical, social and economic access to sufficient, safe, and nutritious food which meets their dietary needs and food preference for an active and health life” [22] and the extended concept of food security. Availability, access, utilization and stability are four pillars proposed by the United Nations Committee on World Food Security (CFS) and FAO, and has been widely recognized as the key pillars since the 2007–08 food crisis [23]. With the in-depth understanding of food security, the HLPE-FSN proposed additional pillars: agency and sustainability, into the food security framework in 2020 [13]. The GOHI-FS framework was based on the extension of the six-dimensional framework. Agency refers to the capacity of individuals or groups to make their decision of food and their involvement in food policies and governance, which relied on the agency capacity to provide policy, finical, and technical supports for food security. Sustainability refers to Natural & Social Circumstance which evaluates the natural, social, and economic long-term sustainability for food generation and adaption. The second-level indicators were organized based on the SPO model proposed by Donabedian [14]. The structures refer to nature, social resources and communities, that provide services, resources, and facilities to support the food systems. Processes refer to attributes (such as interventions, policies) of activities to ensure the functioning well of food security. Outcomes refer to related outcomes of food security. This design facilitates to trace specific structural and process weaknesses when identifying problems in a results-oriented manner, and to inform decisions by the relevant stakeholders. And our third-level indicator focus on perspectives on the One Health spotlight related to human, animal, plant, ecosystem, and social environments for food security.

The One Health concept has gained increased attention and progress from policymakers and scientists in food security [6, 24]. FAO is actively working with partners to develop and implement effective One Health strategies. Priorities for improving the capacity against food insecurity include developing early warning systems on animal and plant diseases, biosecurity for animals and plants disease management, AMR risk management and enhancing One Health systems [9]. In the report of global strategy for food safety 2022–2030, WHO proposed the One Health approach for emerging diseases and hazards detection and control for food safety improvement [25]. The Quadripartite (FAO, UNEP, WHO, and WOAH) released its first One Health Joint Plan of Action (2022–2026), which emphasizes specific One Health actions to address risks in food safety, including strengthening food control systems and food safety coordination, enhancing foodborne disease surveillance, and improving data surveillance and analysis for risk management in the food systems [26]. The methodology in developing GOHI-FS followed the process of well-known assessment tools, such as GFSI, FSI, SDG Index and HDI. GOHI-FS, which broadly collected data from authoritative sources, constructed according to the latest conceptual framework, and assessed by the standard process with agreement by expert advisory committee, can be used to identify gaps and weaknesses in the food systems for countries, and promote the implementation of One Health approaches for achieving food security. It can also be a supplementary tool for the Quadripartite to track the progress of achieving proposed One Health actions, facilitating evidence-based policy and practice of One Health.

### Findings from the pilot results

Findings from our pilot analysis suggested clear gaps across countries/territories and regions, including disparities in food supply and demand, lack of clear actions for guiding food safety, lack of sustainability environment on natural and social resources, and lack of sufficient capacity building from government or agency. Specifically, for Food Supply and Demand, although some countries/territories in Europe and North America, such as the US, Greece and Turkey showed better performance of this domain than other countries, they also showed significant worse performance on food loss and waste (*P* < 0.05 by ANOVA). It highlights the urgency for those countries/territories to reduce food waste that can further minimize the burden of agriculture on climate, soils, water, atmosphere, and biodiversity and facilitate a sustainable food system. Political and legislative interventions should be enhanced to control food loss and waste [27]. Sub-Saharan Africa and South Asia showed low performance on food safety with a high burden of foodborne illness burden. In contrast, Europe and Central Asia and East Asia and Pacific had higher scores on foodborne illness burden. The reason may be those countries/territories have relatively mature food safety surveillance and recall systems than less-developed regions [28]. In addition, although most of countries/territories have their own governance systems to control or monitor food safety, it still lacks unified and assessable criteria to distinguish country performance with high granularity through publicly available sources. Data used in our indicators were limited to the use of binary (Yes/No) or five-level ordered data (i.e., food safety score reported by WHO), which might not sufficiently reveal the real performance. Similar problems also occurred in some indicators such as nutrition promotion. Further efforts are warranted to detail and refine the evaluation criteria with a multiple-level categorical measurement.

Our results found an overall low performance in Government Support and Response globally. For one reason, data for understanding the national differences on agriculture education programme were largely absent in many countries/territories. For another, it may indeed reflect the lack of agency support. Agriculture training programme is important to facilitate food systems transformation, which requires scientific technology and well-educated human resources in response to food insecurity risks [29]. The indicator was measured by the percentage of tertiary graduates from agriculture programmes. However, relevant data was largely absent and out of date, so we removed the score from the final total score. The absence of data also reflects inadequate capacity building from agency level in response to food insecurity.

Overall, North American showed on average better performance in all five dimensions of GOHI-FS, while sub-Saharan Africa had overall low performance of these dimensions. The regional heterogeneity of GOHI-FS might be attributed to notable heterogeneity of nature resources and socioeconomic developments. The score of GOHI-FS showed high correlations with economic indicators such as GPD per capita, social development indicators such as SDI, health indictors such as health expenditure and life expectancy, suggesting the importance of double down on catch-up development. Appropriate policies or strategies for stimulating economic growth, ensuring education attainment, and improving the status of women can be set as priorities not only for the country’s development, but important pathways to achieve food security. In addition, more financial, human resources, and technology investments are needed to reinforce the top-level design, promote digital agriculture and the transformation of the food system, which can lead to increased effectiveness and adaption on healthy food systems, and improve regional food security.

### Limitations

Some limitations should be noted for GOHI-FS. First, to ensure the quality of global data, most of the data were retrieved from international authoritative agencies. However, the overall data missing rate is 19.4% and commonly occurred in some developing countries/territories, which may pose a challenge to precisely evaluate the performance of food security in those countries/territories. To minimize the impact of missing data, we adopted composited factors (health and social development data) to interpolate the missing data. We also tried interpolation by single variable (i.e., GDP, EXP), and the results did not change our main conclusions. It should be noted the data we retrieved in 2020 that reflected the situations in 2019 or earlier since part of data had a long updating interval. Thus, the index didn’t reflect the negative impact of COVID-19 pandemic on food security [30]. Second, the weighting scheme was mainly determined by our expert committee the FAHP approach. According to previous experience of other evaluation tools, it may be hard to achieve a broader consensus about the weights. Thus, some of them also adopted equal weights for each level indicator [31]. Further efforts should provide alternative weighting scheme with more objective approaches and investigate the robustness of the results by different weighting schemes. Third, GOHI provides an evaluation tool for One Health performance across countries/territories. To ensure the independence of indicators and avoid overlap across different aspects, GOHI has been constructed with a coordinated indicator framework that evaluates each aspect once although such an aspect may be a common problem in the One Health interface. For example, health, zoonotic diseases and antimicrobial resistance in livestock were measured in the GOHI sub-index Intrinsic Drivers Index [32], zoonoses [33] and AMR [34], respectively. Those issues are also associated with food security while we did not repeatedly include them in the GOHI-FS framework. Further efforts should focus on the optimization of the calculation and indicator framework to balance conceptual completeness and model applicability between the whole GOHI framework and its sub-index framework. Last, due to the data availability, GOHI-FS temporarily applies to national levels only. Further studies are warranted to extend its application at subregional levels and make appropriate modifications based on local context.

## Conclusions

Food insecurity has been on the rise worldwide. With the growing recognition that achieving food security requires positive actions between the human–animal-environment interfaces, we developed GOHI-FS, a part of the global One Health index framework, with three-level indicator framework and used global authoritative data to evaluate the performance of food security under the One Health concept around the world. According to our pilot analysis, more actions are warranted to promote government support and response globally. In addition, sub-Saharan Africa needs more actions to alleviate its severe food insecurity.

The proposed GOHI-FS is an initial progress to understand current performance on food security under the One Health concept. The index should be continuously verified, revised, and improved. Future efforts are expected to incorporate countries/territories to improve the data completeness and identify the key points for implement interventions to promote the overall health on human, animal, plants, and environment, and achieve sustainable food systems for food security.

### Supplementary Information

**Additional file 1.** Data sources of three-level indicators of GOHI-FS.

**Additional file 2.** Regional categories of 146 countries/territories by World Bank standard country coding for 7 world regions.

**Additional file 3.** Countries/territories grouped by Social-Demographic Index.

**Additional file 4.** Spearman correlation coefficients across second-level indicators of GOHI-FS.

**Additional file 5.** Spearman correlation coefficients across third-level indicators of GOHI-FS.

**Additional file 6.** Performance of GOHI-FS across countries/territories.

**Additional file 7.** Global distribution of the total score of GOHI-FS.

## Data Availability

The full study protocol and the datasets, are available, following manuscript publication, upon request from the corresponding author.

## Abbreviations

AMR: Antimicrobial resistance
CHE: Current health expenditure
COVID-19: Coronavirus disease 2019
EXP: Life expectancy
FAHP: Fuzzy Analytical Hierarchy Process
FAO: Food and Agriculture Organization of the United Nations
FSI: Food sustainability index
GBD: Global Burden of Disease
GFSI: Global food security index
GHI: Global hunger index
GDP: Gross Domestic Product
GOHI: Global One Health Index
GOHI -FS: Global One Health Index-Food Security
HLPE-FSN: High-Level Panel of Experts on Food Security and Nutrition
OECD: Organization for Economic Cooperation and Development
SDI: Social-Demographic Index
WOAH: World Organization for Animal Health
OHHLEP: One Health High Level Expert Panel
SDGs: Sustainable Development Goals
UNEP: United Nations Environment Programme
WHO: World Health Organization
