# Supplementary material for: Assessing food security performance from the One Health concept: an evaluation tool based on the Global One Health Index
Source: Infect Dis Poverty. 2023 Sep 22;12:88. doi: 10.1186/s40249-023-01135-7 (PMC10514978; doi:10.1186/s40249-023-01135-7)
Supplement: Supplementary file 1 — Additional file 1. Data sources of three-level indicators of GOHI-FS. [file 40249_2023_1135_MOESM1_ESM.docx]

#

# **Additional file 1:** Data sources of three-level indicators of GOHI-FS

| Code | Indicators | Data source | Download Link |
| --- | --- | --- | --- |
| 1.1.1 | Ratio of population growth | UN | [http://data.un.org/Data.?d=PopDiv&f=variableID%3a47](http://data.un.org/Data.aspx?d=PopDiv&f=variableID:47) |
| 1.1.2 | Ratio of refugees and internally displaced people | UNHCR | <https://www.unhcr.org/refugee-statistics/download/?url=Xm6zSN> |
| 1.1.3 | Ratio of moderately or severely food insecure people | FAO | <https://www.fao.org/faostat/en/#data/FS> |
| 1.2.1 | Food loss | FAO | <http://www.fao.org/faostat/en/#data/FBS> |
| 1.2.2 | Food waste | UNEP | <https://www.unep.org/resources/report/unep-food-waste-index-report-2021> |
| 1.3.1 | Logistic performance index | World Bank | <https://lpi.worldbank.org/international/global> |
| 1.3.2 | Net capital stocks | FAO | <https://www.fao.org/faostat/en/#data/CS> |
| 1.3.3 | Percent of arable land equipped for irrigation | FAO | <https://www.fao.org/faostat/en/#data/FS> |
| 1.4.1 | Cereal import dependency ratio | FAO | <https://www.fao.org/faostat/en/#data/FS> |
| 1.4.2 | Value of food imports over total merchandise exports | FAO | [https://www.fao.org/faostat/en/#data/FS](#data/FS) |
| 1.4.3 | Food aid | FAO | <https://www.fao.org/faostat/en/#data/FA> |
| 1.5.1 | Average value of food production | FAO | <https://www.fao.org/faostat/en/#data/QV> |
| 1.5.2 | Food production viability | FAO | [https://www.fao.org/faostat/en/#data/FS](#data/FS) |
| 2.1.1 | Food safety agency | self-designed | <https://www.fao.org/faolex/country-profiles/en/> |
| 2.1.2 | Food policy, legal and regulatory framework | self-designed | <https://www.fao.org/faolex/country-profiles/en/> |
| 2.2.1 | Inspections in farm-to-fork food chain | self-designed | <https://www.fao.org/faolex/country-profiles/en/> |
| 2.2.2 | Food recalls | self-designed | <https://www.fao.org/faolex/country-profiles/en/> |
| 2.3.1 | Food safety score | WHO | <https://www.who.int/data/gho/data/indicators/indicator-details/GHO/food-safety-spar> |
| 2.4.1 | DALYs (Disability-Adjusted Life Years) of diarrhea | GBD | <https://vizhub.healthdata.org/gbd-results/> |
| 3.1.1 | Average dietary energy supply adequacy | FAO | [https://www.fao.org/faostat/en/#data/FS](https://www.fao.org/faostat/en/) |
| 3.1.2 | Average protein supply (g/cap/day) (3-year average) | FAO | [https://www.fao.org/faostat/en/#data/FS](https://www.fao.org/faostat/en/) |
| 3.1.3 | Per capita food supply variability (kcal/cap/day) | FAO | [https://www.fao.org/faostat/en/#data/FS](https://www.fao.org/faostat/en/) |
| 3.2.1 | Nutrition labeling | FAO | <https://www.fao.org/fao-who-codexalimentarius/about-codex/members/zh/> |
| 3.2.2 | Nutrition guideline | FAO | <https://www.fao.org/nutrition/education/food-dietary-guidelines/home/zh/> |
| 3.2.3 | Nutrition education programme | WHO | <https://www.who.int/data/gho/data/indicators/indicator-details/GHO/existence-of-operational-policy-strategy-action-plan-to-reduce-unhealthy-diet-related-to-ncds-> |
| 3.3.1 | Undernourishment | FAO | [https://www.fao.org/faostat/en/#data/FS](https://www.fao.org/faostat/en/) |
| 3.3.2 | Stunting in children under five | FAO | [https://www.fao.org/faostat/en/#data/FS](https://www.fao.org/faostat/en/) |
| 3.3.3 | Anemia among women of reproductive age | FAO | [https://www.fao.org/faostat/en/#data/FS](https://www.fao.org/faostat/en/) |
| 4.1.1 | Food affected by extreme weather conditions, disasters or crisis | FEWS NET | [https://fews.net/fews-data/333?tid=All&field_data_portal_date_start%5Bvalue%5D%5Byear%5D=2020&field_data_portal_date_start](https://fews.net/fews-data/333?tid=All&field_data_portal_date_start%5bvalue%5d%5byear%5d=2020&field_data_portal_date_start) |
| 4.2.1 | Per person land under cereal production | World Bank | <https://data.worldbank.org/indicator/AG.LND.ARBL.HA.PC?view=chart> |
| 4.2.2 | Agricultural water withdrawal as % of total renewable water resources | World Bank | <https://datacatalog.worldbank.org/search/dataset/0037712> |
| 4.2.3 | Agriculture area under organic agric | FAO | [https://www.fao.org/faostat/en/#data/EL](https://www.fao.org/faostat/en/) |
| 4.2.4 | Naturally regenerating forest | FAO | [https://www.fao.org/faostat/en/#data/EL](https://www.fao.org/faostat/en/) |
| 4.2.5 | Manure management | FAO | [https://www.fao.org/faostat/en/#data/EMN](https://www.fao.org/faostat/en/) |
| 4.3.1 | Trade balance indicators | UN | <https://unctadstat.unctad.org/wds/TableViewer/tableView.aspx> |
| 4.3.2 | Economic vulnerability index | UN | <https://www.un.org/development/desa/dpad/least-developed-country-category/ldc-data-retrieval.html> |
| 4.4.1 | Agriculture value added per worker | FAO | [https://www.fao.org/faostat/en/#data/OE](https://www.fao.org/faostat/en/) |
| 4.5.1 | Agricultural import tariffs | WTO | <https://stats.wto.org/> |
| 4.5.2 | Consumer prices food indices | FAO | [https://www.fao.org/faostat/en/#data/CP](https://www.fao.org/faostat/en/) |
| 4.5.3 | Food price inflation | FAO | [https://www.fao.org/faostat/en/#data/CP](https://www.fao.org/faostat/en/) |
| 5.1.1 | Government investment on agriculture | FAO | [https://www.fao.org/faostat/en/#data/IG](https://www.fao.org/faostat/en/) |
| 5.1.2 | Credit to agriculture, forestry, fishing | FAO | [https://www.fao.org/faostat/en/#data/IC](https://www.fao.org/faostat/en/) |
| 5.1.3 | R&D expenditures | FAO | [https://www.fao.org/faostat/en/#search/Agriculture%20research%20spending](https://www.fao.org/faostat/en/) |
| 5.2.1 | Training programme | UN | [http://data.un.org/Data.aspx?q=agriculture&d=UNESCO&f=series%3aFOSGP_56_F600](http://data.un.org/Data.aspx?q=agriculture&d=UNESCO&f=series:FOSGP_56_F600) |
| 5.2.2 | Smart and digital agriculture | IAEA | <https://www.iaea.org/projects/technical-cooperation-projects?topics=3029&status=3723&combine=> |
