# Supplementary material for: Assessing food security performance from the One Health concept: an evaluation tool based on the Global One Health Index
Source: Infect Dis Poverty. 2023 Sep 22;12:88. doi: 10.1186/s40249-023-01135-7 (PMC10514978; doi:10.1186/s40249-023-01135-7)
Supplement: Supplementary file 2 — Additional file 2. Regional categories of 146 countries/territories by World Bank standard country coding for 7 world regions. [file 40249_2023_1135_MOESM2_ESM.docx]

# **Additional file 2:** Regional categories of 146 countries/territories by World Bank standard country coding for 7 world regions

| Regions | country |
| --- | --- |
| East Asia and Pacific | Australia, Brunei Darussalam, Cambodia, China, Fiji, Indonesia, Japan, Laos, Malaysia, Mongolia, Myanmar, New Zealand, Papua New Guinea, Philippines, Singapore, South Korea, Thailand, Timor-Leste, Viet Nam |
| Europe and Central Asia | Albania, Armenia, Austria, Azerbaijan, Belarus, Belgium, Bulgaria, Croatia, Cyprus, Czech Republic, Denmark, Estonia, Finland, France, Georgia, Germany, Greece, Hungary, Iceland, Ireland, Italy, Kazakhstan, Kyrgyzstan, Latvia, Lithuania, Luxembourg, Moldova, Montenegro, Netherlands, North Macedonia, Norway, Poland, Portugal, Romania, Russia, Serbia, Slovakia, Slovenia, Spain, Sweden, Switzerland, Tajikistan, Turkey, Turkmenistan, Ukraine, United Kingdom, Uzbekistan |
| Latin America and the Caribbean | Argentina, Belize, Bolivia, Brazil, Barbados, Chile, Colombia, Costa Rica, Cuba, Dominican Republic, Ecuador, Honduras, Mexico, Nicaragua, Paraguay, Peru, Trinidad and Tobago, Uruguay |
| Middle East and North Africa | Algeria, Bahrain, Egypt, Iran, Iraq, Israel, Jordan, Lebanon, Libya, Malta, Morocco, Oman, Qatar, Saudi Arabia, Tunisia, United Arab Emirates |
| North America | Canada, United States of America |
| South Asia | Afghanistan, Bangladesh, Bhutan, India, Nepal, Pakistan, Sri Lanka |
| Sub-Saharan Africa | Benin, Botswana, Burkina Faso, Burundi, Cabo Verde, Cameroon, Central African Republic, Chad, Cote d'Ivoire, Dem. Rep. Congo, Ethiopia, Gabon, Ghana, Guinea, Kenya, Lesotho, Liberia, Madagascar, Malawi, Mali, Mauritania, Mauritius, Mozambique, Namibia, Niger, Nigeria, Rwanda, Senegal, Seychelles, Sierra Leone, South Africa, Sudan, Tanzania, Togo, Uganda, Zambia, Zimbabwe |
