# Supplementary material for: Assessing food security performance from the One Health concept: an evaluation tool based on the Global One Health Index
Source: Infect Dis Poverty. 2023 Sep 22;12:88. doi: 10.1186/s40249-023-01135-7 (PMC10514978; doi:10.1186/s40249-023-01135-7)
Supplement: Supplementary file 3 — Additional file 3. Countries/territories grouped by Social-Demographic Index. [file 40249_2023_1135_MOESM3_ESM.docx]

#

# **Additional file 3:** Countries/territories grouped by Social-Demographic Index

| SDI-quintile | lower-upper bound | country |
| --- | --- | --- |
| High SDI | 0.805129 - 1 | Australia, Austria, Belgium, Brunei Darussalam, Canada, Cyprus, Czech Republic, Denmark, Estonia, Finland, France, Germany, Ireland, Iceland, Japan, South Korea, Lithuania, Luxembourg, Latvia, Netherlands, Norway, New Zealand, Qatar, Singapore, Slovakia, Slovenia, Sweden, Switzerland, United Arab Emirates, United Kingdom, United States of America |
| High-middle SDI | 0.689504 - 0.805129 | Argentina, Bulgaria, Bahrain, Belarus, Barbados, Chile, Spain, Georgia, Greece, Croatia, Hungary, Israel, Italy, Jordan, Kazakhstan, Lebanon, Libya, Sri Lanka, Moldova, North Macedonia, Malta, Montenegro, Mauritius, Malaysia, Oman, Poland, Portugal, Romania, Russia, Saudi Arabia, Serbia, Seychelles, Trinidad and Tobago, Turkey, Ukraine, Uruguay |
| Middle SDI | 0.607679 - 0.689504 | Albania, Armenia, Azerbaijan, Brazil, Botswana, China, Colombia, Costa Rica, Cuba, Algeria, Ecuador, Egypt, Fiji, Gabon, Indonesia, Iran, Iraq, Mexico, Namibia, Peru, Philippines, Paraguay, Thailand, Turkmenistan, Tunisia, Uzbekistan, Viet Nam, South Africa |
| Low-middle SDI | 0.454743 - 0.607679 | Bangladesh, Belize, Bolivia, Bhutan, Cameroon, Cabo Verde, Dominican Republic, Ghana, Honduras, India, Kenya, Kyrgyzstan, Cambodia, Laos, Lesotho, Morocco, Myanmar, Mongolia, Mauritania, Nigeria, Nicaragua, Sudan, Tajikistan, Timor-Leste, Zambia, Zimbabwe |
| Low SDI | 0 - 0.454743 | Afghanistan, Burundi, Benin, Burkina Faso, Central African Republic, Cote d'Ivoire, Dem. Rep. Congo, Ethiopia, Guinea, Liberia, Madagascar, Mali, Mozambique, Malawi, Niger, Nepal, Pakistan, Papua New Guinea, Rwanda, Senegal, Sierra Leone, Chad, Togo, Tanzania, Uganda |
