# Supplementary material for: Assessing food security performance from the One Health concept: an evaluation tool based on the Global One Health Index
Source: Infect Dis Poverty. 2023 Sep 22;12:88. doi: 10.1186/s40249-023-01135-7 (PMC10514978; doi:10.1186/s40249-023-01135-7)

#

# **Additional file 4:** Spearman correlation coefficients across second-level indicators of GOHI-FS.


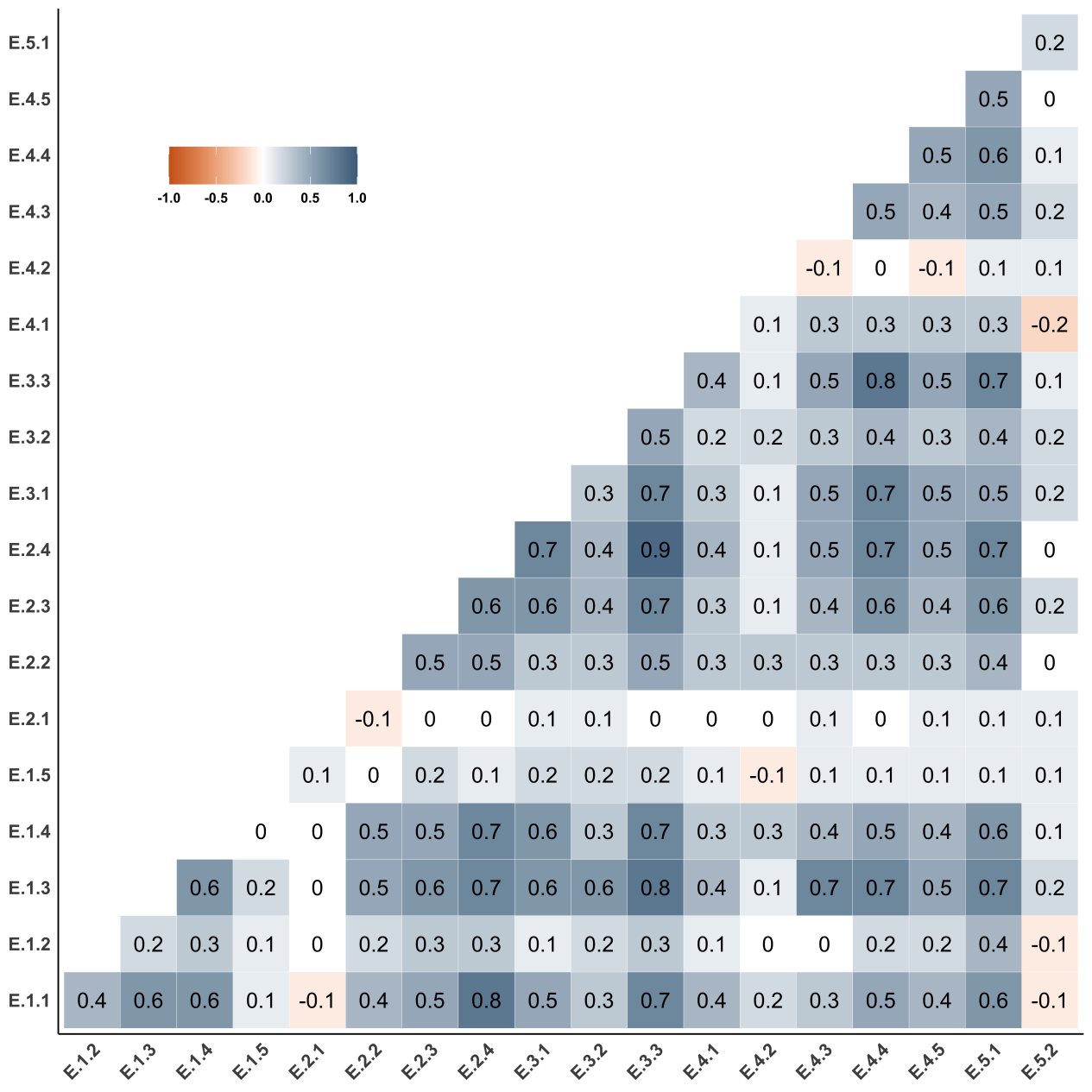

Supplement: Supplementary file 4 — Additional file 4. Spearman correlation coefficients across second-level indicators of GOHI-FS. [file 40249_2023_1135_MOESM4_ESM.docx]
