# Supplementary figures and images for: Assessing food security performance from the One Health concept: an evaluation tool based on the Global One Health Index
Source: Infect Dis Poverty. 2023 Sep 22;12:88. doi: 10.1186/s40249-023-01135-7 (PMC10514978; doi:10.1186/s40249-023-01135-7)

#

# **Additional file 5:** Spearman correlation coefficients across third-level indicators of GOHI-FS.

**
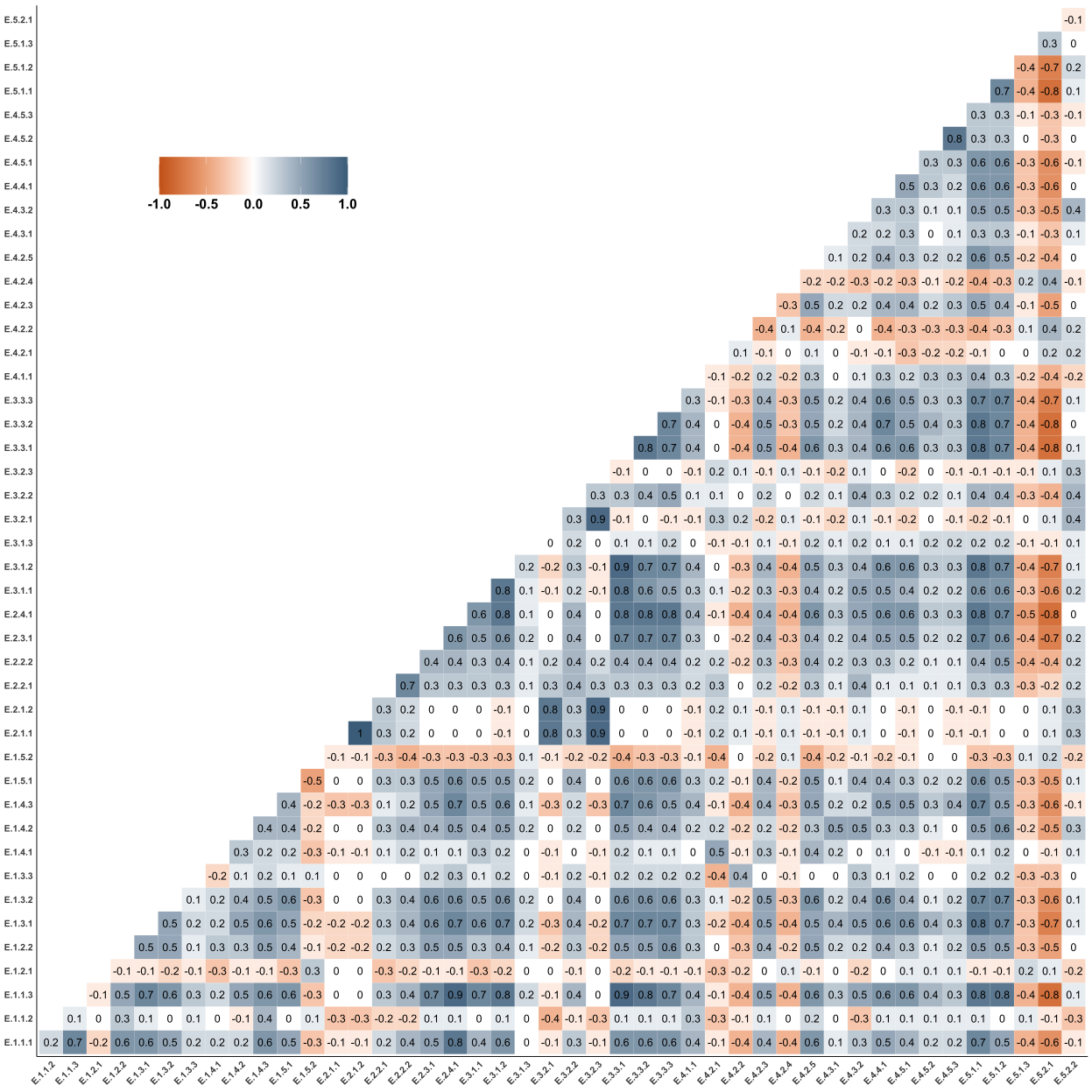
**

Supplement: Supplementary file 5 — Additional file 5. Spearman correlation coefficients across third-level indicators of GOHI-FS. [file 40249_2023_1135_MOESM5_ESM.docx]
