# Supplementary material for: Assessing food security performance from the One Health concept: an evaluation tool based on the Global One Health Index
Source: Infect Dis Poverty. 2023 Sep 22;12:88. doi: 10.1186/s40249-023-01135-7 (PMC10514978; doi:10.1186/s40249-023-01135-7)
Supplement: Supplementary file 6 — Additional file 6. Performance of GOHI-FS across countries/territories. [file 40249_2023_1135_MOESM6_ESM.docx]

# **Additional file 6:** Performance of GOHI-FS across countries/territories.

Countries/territories are listed in alphabetical order and are divided into five ranking groups.

| Countries/  territories | SDI category | Food Demand & Supply | Food Safety | Nutrition | Natural & social circumstances | Government support & response | Total |
| --- | --- | --- | --- | --- | --- | --- | --- |
| Afghanistan | Low SDI | 37.2 | 38.0 | 51.4 | 31.7 | 18.2 | 35.3 |
| Albania | Middle SDI | 58.9 | 88.5 | 86.9 | 63.6 | 15.8 | 62.7 |
| Algeria | Middle SDI | 46.3 | 73.4 | 69.9 | 60.8 | 26.3 | 55.3 |
| Argentina | High-middle SDI | 61.8 | 90.4 | 87.9 | 71.4 | 44.4 | 71.2 |
| Armenia | Middle SDI | 51.5 | 64.1 | 74.3 | 62.6 | 19.6 | 54.4 |
| Australia | High SDI | 71.6 | 99.1 | 91.5 | 80.4 | 50.3 | 78.6 |
| Austria | High SDI | 70.7 | 98.4 | 95.4 | 73.5 | 45.1 | 76.6 |
| Azerbaijan | Middle SDI | 58.3 | 87.4 | 62.9 | 57.8 | 23.4 | 58.0 |
| Bahrain | High-middle SDI | 57.3 | 60.9 | 69.0 | 49.5 | 24.1 | 52.1 |
| Bangladesh | Low-middle SDI | 58.5 | 49.2 | 58.6 | 54.3 | 37.8 | 51.7 |
| Barbados | High-middle SDI | 70.6 | 61.5 | 81.1 | 65.9 | 21.0 | 60.0 |
| Belarus | High-middle SDI | 61.6 | 97.2 | 71.0 | 62.1 | 34.3 | 65.3 |
| Belgium | High SDI | 67.4 | 94.5 | 93.4 | 73.2 | 39.0 | 73.5 |
| Belize | Low-middle SDI | 59.5 | 72.7 | 74.0 | 62.7 | 26.3 | 59.0 |
| Benin | Low SDI | 37.3 | 53.3 | 64.1 | 58.4 | 36.3 | 49.9 |
| Bhutan | Low-middle SDI | 55.9 | 50.0 | 55.0 | 62.4 | 32.4 | 51.1 |
| Bolivia | Low-middle SDI | 59.0 | 55.4 | 69.9 | 62.4 | 28.0 | 55.0 |
| Botswana | Middle SDI | 38.9 | 69.7 | 45.4 | 58.7 | 39.2 | 50.4 |
| Brazil | Middle SDI | 60.8 | 92.9 | 86.5 | 65.7 | 40.0 | 69.2 |
| Brunei Darussalam | High SDI | 68.2 | 67.4 | 77.2 | 73.9 | 28.3 | 63.0 |
| Bulgaria | High-middle SDI | 62.5 | 92.4 | 76.9 | 68.1 | 32.9 | 66.6 |
| Burkina Faso | Low SDI | 35.3 | 57.4 | 51.5 | 55.0 | 32.9 | 46.4 |
| Burundi | Low SDI | 28.1 | 36.1 | 39.1 | 25.3 | 26.7 | 31.0 |
| Cabo Verde | Low-middle SDI | 52.1 | 70.3 | 60.1 | 58.1 | 20.1 | 52.1 |
| Cambodia | Low-middle SDI | 51.6 | 65.6 | 63.8 | 58.7 | 35.1 | 55.0 |
| Cameroon | Low-middle SDI | 35.0 | 36.8 | 58.7 | 37.1 | 30.2 | 39.6 |
| Canada | High SDI | 64.2 | 94.6 | 88.3 | 78.8 | 51.6 | 75.5 |
| Central African Republic | Low SDI | 28.7 | 30.3 | 25.1 | 54.5 | 9.1 | 29.5 |
| Chad | Low SDI | 31.0 | 30.3 | 41.6 | 29.3 | 16.0 | 29.6 |
| Chile | High-middle SDI | 71.9 | 82.0 | 88.2 | 64.4 | 38.3 | 68.9 |
| China | Middle SDI | 72.0 | 91.8 | 91.1 | 63.6 | 54.9 | 74.7 |
| Colombia | Middle SDI | 55.0 | 74.5 | 67.3 | 60.6 | 31.1 | 57.7 |
| Costa Rica | Middle SDI | 55.7 | 88.0 | 80.1 | 69.2 | 46.3 | 67.9 |
| Cote d'Ivoire | Low SDI | 42.9 | 34.8 | 53.7 | 66.9 | 24.7 | 44.6 |
| Croatia | High-middle SDI | 60.1 | 91.3 | 79.1 | 71.0 | 32.8 | 66.9 |
| Cuba | Middle SDI | 57.0 | 85.6 | 79.2 | 58.4 | 28.1 | 61.7 |
| Cyprus | High SDI | 55.3 | 96.8 | 74.8 | 77.5 | 30.0 | 66.9 |
| Czech Republic | High SDI | 68.8 | 84.5 | 60.5 | 69.7 | 39.5 | 64.6 |
| Dem. Rep. Congo | Low SDI | 33.1 | 33.5 | 38.9 | 56.3 | 11.0 | 34.6 |
| Denmark | High SDI | 68.2 | 94.8 | 94.5 | 80.1 | 41.0 | 75.7 |
| Dominican Republic | Low-middle SDI | 58.7 | 71.1 | 73.5 | 68.8 | 18.1 | 58.0 |
| Ecuador | Middle SDI | 57.5 | 86.3 | 60.5 | 64.0 | 33.1 | 60.3 |
| Egypt | Middle SDI | 50.5 | 67.1 | 67.7 | 63.9 | 22.4 | 54.3 |
| Estonia | High SDI | 68.6 | 89.9 | 85.4 | 74.4 | 34.8 | 70.6 |
| Ethiopia | Low SDI | 39.1 | 37.7 | 49.2 | 36.5 | 35.9 | 39.7 |
| Fiji | Middle SDI | 57.9 | 52.8 | 70.3 | 63.3 | 40.3 | 56.9 |
| Finland | High SDI | 68.9 | 99.5 | 96.0 | 76.2 | 36.2 | 75.4 |
| France | High SDI | 67.3 | 92.4 | 90.4 | 77.0 | 49.0 | 75.2 |
| Gabon | Middle SDI | 52.2 | 51.6 | 50.9 | 55.4 | 22.5 | 46.5 |
| Georgia | High-middle SDI | 48.8 | 79.2 | 68.4 | 54.7 | 15.8 | 53.4 |
| Germany | High SDI | 70.7 | 90.1 | 93.0 | 75.2 | 48.4 | 75.5 |
| Ghana | Low-middle SDI | 46.3 | 42.3 | 58.2 | 61.9 | 28.1 | 47.3 |
| Greece | High-middle SDI | 56.8 | 94.4 | 90.6 | 76.0 | 33.6 | 70.3 |
| Guinea | Low SDI | 32.2 | 53.5 | 49.6 | 51.2 | 20.6 | 41.4 |
| Honduras | Low-middle SDI | 47.9 | 81.0 | 69.3 | 60.5 | 17.5 | 55.3 |
| Hungary | High-middle SDI | 64.6 | 88.8 | 80.9 | 71.2 | 27.0 | 66.5 |
| Iceland | High SDI | 68.2 | 68.5 | 94.6 | 77.0 | 38.8 | 69.4 |
| India | Low-middle SDI | 64.8 | 73.5 | 57.3 | 61.1 | 43.5 | 60.0 |
| Indonesia | Middle SDI | 65.4 | 62.4 | 67.4 | 64.2 | 40.3 | 59.9 |
| Iran | Middle SDI | 55.8 | 60.8 | 76.3 | 60.8 | 35.6 | 57.9 |
| Iraq | Middle SDI | 34.4 | 48.3 | 42.3 | 63.5 | 26.0 | 42.9 |
| Ireland | High SDI | 65.2 | 98.4 | 97.3 | 76.1 | 43.8 | 76.2 |
| Israel | High-middle SDI | 61.9 | 64.9 | 95.6 | 74.2 | 40.9 | 67.5 |
| Italy | High-middle SDI | 73.4 | 100.0 | 89.5 | 77.1 | 49.0 | 77.8 |
| Japan | High SDI | 84.7 | 100.0 | 83.3 | 69.6 | 48.6 | 77.2 |
| Jordan | High-middle SDI | 48.9 | 62.1 | 58.2 | 66.4 | 18.9 | 50.9 |
| Kazakhstan | High-middle SDI | 60.2 | 90.4 | 72.3 | 67.4 | 25.7 | 63.2 |
| Kenya | Low-middle SDI | 39.9 | 44.7 | 58.8 | 38.0 | 37.7 | 43.8 |
| Kyrgyzstan | Low-middle SDI | 55.4 | 75.2 | 61.8 | 56.8 | 13.4 | 52.5 |
| Laos | Low-middle SDI | 54.2 | 43.7 | 56.2 | 63.2 | 26.9 | 48.9 |
| Latvia | High SDI | 63.0 | 90.7 | 87.6 | 70.5 | 26.8 | 67.7 |
| Lebanon | High-middle SDI | 49.1 | 54.8 | 61.5 | 61.0 | 31.2 | 51.5 |
| Lesotho | Low-middle SDI | 41.5 | 52.3 | 38.1 | 47.3 | 27.4 | 41.3 |
| Liberia | Low SDI | 36.4 | 37.9 | 40.3 | 47.8 | 10.9 | 34.7 |
| Libya | High-middle SDI | 53.7 | 55.0 | 55.6 | 48.7 | 21.2 | 46.8 |
| Lithuania | High SDI | 63.3 | 82.6 | 83.7 | 71.0 | 32.7 | 66.7 |
| Luxembourg | High SDI | 63.3 | 96.0 | 83.6 | 80.8 | 45.1 | 73.8 |
| Madagascar | Low SDI | 41.5 | 35.9 | 36.0 | 33.8 | 30.9 | 35.6 |
| Malawi | Low SDI | 32.5 | 33.8 | 54.2 | 30.7 | 26.8 | 35.6 |
| Malaysia | High-middle SDI | 59.8 | 60.5 | 75.0 | 69.7 | 39.1 | 60.8 |
| Mali | Low SDI | 32.5 | 64.1 | 56.5 | 60.6 | 28.3 | 48.4 |
| Malta | High-middle SDI | 53.0 | 94.4 | 94.1 | 73.1 | 29.7 | 68.9 |
| Mauritania | Low-middle SDI | 41.6 | 32.2 | 60.7 | 62.6 | 16.8 | 42.8 |
| Mauritius | High-middle SDI | 70.1 | 62.8 | 70.5 | 64.0 | 45.0 | 62.5 |
| Mexico | Middle SDI | 52.7 | 74.2 | 82.1 | 66.1 | 34.4 | 61.9 |
| Moldova | High-middle SDI | 58.0 | 56.4 | 58.3 | 58.8 | 18.0 | 49.9 |
| Mongolia | Low-middle SDI | 56.1 | 60.8 | 79.1 | 58.7 | 25.3 | 56.0 |
| Montenegro | High-middle SDI | 60.9 | 59.3 | 77.5 | 65.0 | 24.8 | 57.5 |
| Morocco | Low-middle SDI | 53.4 | 57.8 | 73.5 | 68.0 | 42.4 | 59.0 |
| Mozambique | Low SDI | 30.5 | 66.8 | 33.6 | 31.6 | 23.3 | 37.2 |
| Myanmar | Low-middle SDI | 50.7 | 51.5 | 61.4 | 57.6 | 26.7 | 49.6 |
| Namibia | Middle SDI | 46.1 | 47.3 | 66.3 | 58.0 | 33.5 | 50.2 |
| Nepal | Low SDI | 44.2 | 43.9 | 66.7 | 61.1 | 23.8 | 47.9 |
| Netherlands | High SDI | 75.0 | 92.4 | 92.9 | 76.5 | 44.9 | 76.4 |
| New Zealand | High SDI | 64.5 | 64.4 | 91.5 | 75.9 | 49.0 | 69.1 |
| Nicaragua | Low-middle SDI | 52.0 | 85.1 | 52.3 | 60.4 | 27.5 | 55.5 |
| Niger | Low SDI | 27.1 | 30.3 | 44.7 | 59.2 | 12.5 | 34.8 |
| Nigeria | Low-middle SDI | 41.9 | 36.1 | 58.6 | 31.3 | 26.6 | 38.9 |
| North Macedonia | High-middle SDI | 56.7 | 57.7 | 83.8 | 64.7 | 32.4 | 59.1 |
| Norway | High SDI | 74.5 | 94.9 | 94.6 | 78.8 | 39.8 | 76.5 |
| Oman | High-middle SDI | 53.8 | 59.4 | 72.0 | 60.4 | 28.9 | 54.9 |
| Pakistan | Low SDI | 46.5 | 40.6 | 48.7 | 57.5 | 33.6 | 45.4 |
| Papua New Guinea | Low SDI | 54.7 | 43.3 | 44.3 | 57.0 | 25.2 | 44.9 |
| Paraguay | Middle SDI | 56.0 | 86.2 | 74.3 | 63.8 | 17.8 | 59.6 |
| Peru | Middle SDI | 56.2 | 84.4 | 74.7 | 67.1 | 34.4 | 63.4 |
| Philippines | Middle SDI | 52.8 | 84.0 | 68.4 | 62.7 | 32.1 | 60.0 |
| Poland | High-middle SDI | 67.5 | 75.5 | 89.9 | 63.2 | 34.2 | 66.1 |
| Portugal | High-middle SDI | 67.0 | 93.4 | 91.9 | 69.6 | 34.0 | 71.2 |
| Qatar | High SDI | 65.0 | 61.8 | 83.4 | 65.1 | 36.3 | 62.3 |
| Romania | High-middle SDI | 69.2 | 64.2 | 79.9 | 64.9 | 33.7 | 62.4 |
| Russia | High-middle SDI | 72.2 | 91.0 | 77.3 | 68.0 | 39.6 | 69.6 |
| Rwanda | Low SDI | 29.4 | 53.4 | 43.0 | 49.3 | 16.4 | 38.3 |
| Saudi Arabia | High-middle SDI | 62.3 | 61.3 | 84.1 | 68.5 | 27.6 | 60.8 |
| Senegal | Low SDI | 36.0 | 49.4 | 50.8 | 59.0 | 34.0 | 45.9 |
| Serbia | High-middle SDI | 62.3 | 78.4 | 69.5 | 63.2 | 31.2 | 60.9 |
| Seychelles | High-middle SDI | 66.6 | 61.6 | 82.5 | 59.5 | 30.3 | 60.1 |
| Sierra Leone | Low SDI | 36.4 | 35.4 | 48.4 | 48.3 | 12.3 | 36.2 |
| Singapore | High SDI | 66.7 | 66.7 | 82.8 | 77.1 | 33.7 | 65.4 |
| Slovakia | High SDI | 65.8 | 71.1 | 59.8 | 73.4 | 26.5 | 59.3 |
| Slovenia | High SDI | 63.6 | 93.4 | 88.4 | 73.4 | 42.4 | 72.2 |
| South Africa | Middle SDI | 62.9 | 87.3 | 73.2 | 62.6 | 18.8 | 61.0 |
| South Korea | High SDI | 74.7 | 71.0 | 90.8 | 71.2 | 41.3 | 69.8 |
| Spain | High-middle SDI | 66.2 | 91.6 | 91.7 | 77.9 | 46.3 | 74.7 |
| Sri Lanka | High-middle SDI | 56.8 | 49.8 | 70.5 | 66.9 | 36.0 | 56.0 |
| Sudan | Low-middle SDI | 36.3 | 51.8 | 52.7 | 56.8 | 25.5 | 44.6 |
| Sweden | High SDI | 66.7 | 88.9 | 91.7 | 79.1 | 38.7 | 73.0 |
| Switzerland | High SDI | 72.2 | 96.6 | 90.4 | 80.1 | 53.7 | 78.6 |
| Tajikistan | Low-middle SDI | 47.3 | 75.1 | 53.6 | 54.4 | 7.5 | 47.6 |
| Tanzania | Low SDI | 30.8 | 46.4 | 47.2 | 60.9 | 28.4 | 42.7 |
| Thailand | Middle SDI | 63.7 | 59.8 | 72.7 | 66.0 | 40.9 | 60.6 |
| Timor-Leste | Low-middle SDI | 51.0 | 56.3 | 44.2 | 57.1 | 21.9 | 46.1 |
| Togo | Low SDI | 38.2 | 35.9 | 45.6 | 59.0 | 26.3 | 41.0 |
| Trinidad and Tobago | High-middle SDI | 60.9 | 61.3 | 71.3 | 57.0 | 21.9 | 54.5 |
| Tunisia | Middle SDI | 50.6 | 87.9 | 77.1 | 69.7 | 36.1 | 64.3 |
| Turkey | High-middle SDI | 52.5 | 74.0 | 91.7 | 68.7 | 25.3 | 62.5 |
| Turkmenistan | Middle SDI | 62.8 | 55.6 | 72.2 | 70.0 | 23.3 | 56.8 |
| Uganda | Low SDI | 36.1 | 41.3 | 43.1 | 36.0 | 35.6 | 38.4 |
| Ukraine | High-middle SDI | 60.1 | 87.3 | 65.3 | 58.7 | 13.5 | 57.0 |
| United Arab Emirates | High SDI | 63.3 | 94.4 | 66.2 | 69.0 | 28.0 | 64.2 |
| United Kingdom | High SDI | 69.3 | 98.5 | 92.9 | 67.4 | 46.9 | 75.0 |
| United States of America | High SDI | 64.7 | 96.8 | 96.0 | 76.2 | 50.0 | 76.7 |
| Uruguay | High-middle SDI | 57.1 | 90.0 | 85.1 | 68.6 | 27.0 | 65.5 |
| Uzbekistan | Middle SDI | 62.0 | 59.3 | 67.7 | 60.7 | 29.6 | 55.9 |
| Viet Nam | Middle SDI | 67.6 | 90.2 | 79.2 | 59.6 | 45.0 | 68.3 |
| Zambia | Low-middle SDI | 45.8 | 50.5 | 42.4 | 50.0 | 35.9 | 44.9 |
| Zimbabwe | Low-middle SDI | 45.2 | 53.3 | 43.0 | 21.0 | 33.1 | 39.1 |

| Colors in the table represent rankings of scores and regions of countries/territories. | | | | | | | |
| --- | --- | --- | --- | --- | --- | --- | --- |
| Rankings | 1-30 | 31-60 | 61-90 | 91-120 | 121-146 |  |  |
|  |  |  |  |  |  |  |  |
| Regions | East Asia and Pacific | Europe and Central Asia | Latin America and The Caribbean | Middle East and North Africa | North America | South Asia | Sub-Saharan Africa |
