# Supplementary material for: Assessing food security performance from the One Health concept: an evaluation tool based on the Global One Health Index
Source: Infect Dis Poverty. 2023 Sep 22;12:88. doi: 10.1186/s40249-023-01135-7 (PMC10514978; doi:10.1186/s40249-023-01135-7)
Supplement: Supplementary file 7 — Additional file 7. Global distribution of the total score of GOHI-FS. [file 40249_2023_1135_MOESM7_ESM.docx]

#

# **Additional file 7:** Global distribution of the total score of GOHI-FS.


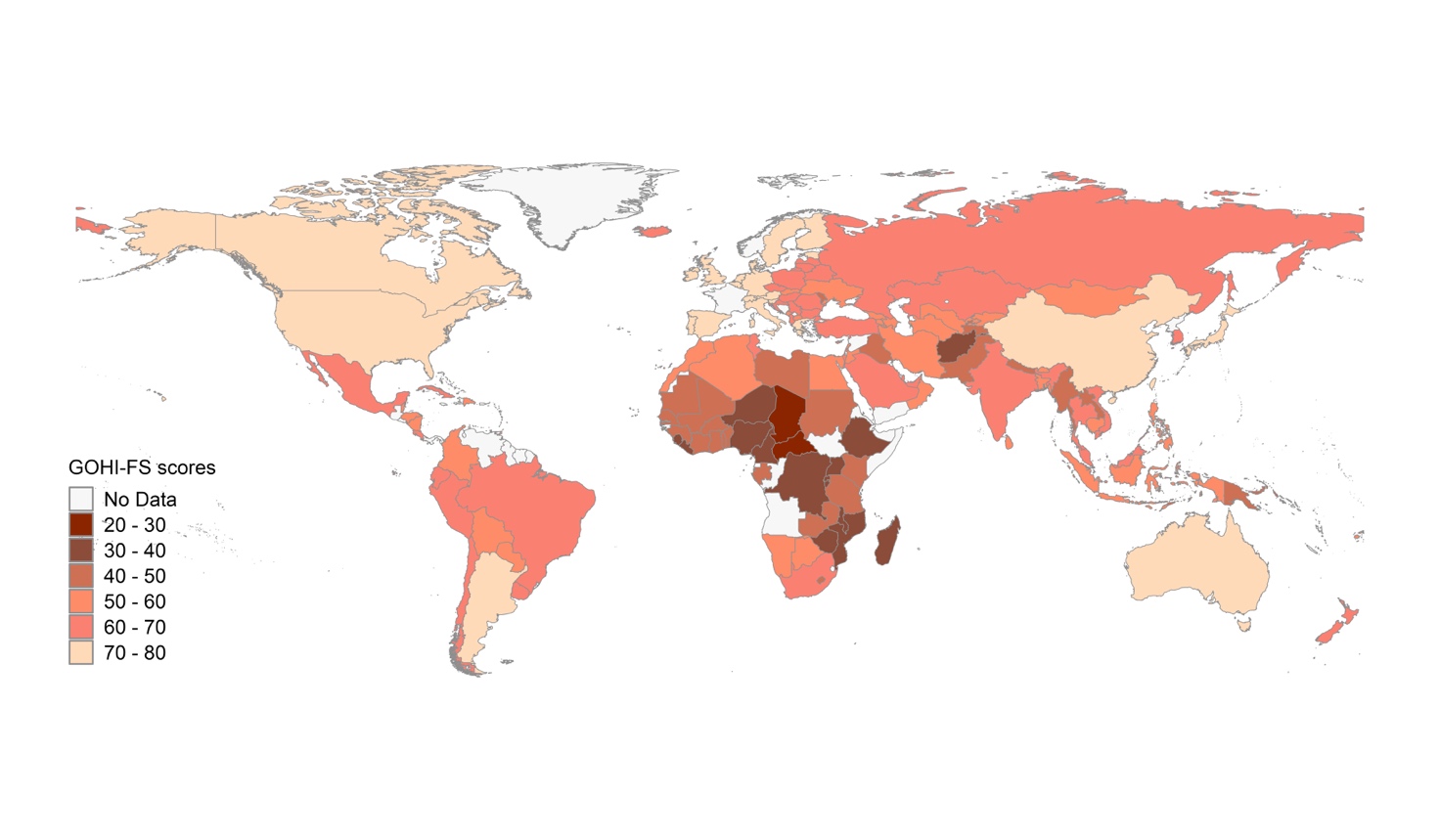
Note: The countries/territories are colored by score range in the map. The map source was from R package ‘rworldmap’. The figure is used to show the global distribution of GOHI-FS scores and does not represent the authors’ position with respect to territorial claims in the map source.
